# Observations on the Epidemic Yellow Fever of Natchez, and of the South-west

**Published:** 1842-06

**Authors:** John W. Monette

**Affiliations:** Washington, Mississippi


### WESTERN JOURNAL

OF

### MEDICINE AND SURGERY.

JUNE, 1842.

Art. I.—*Observations on the epidemic Yellow Fever of Natchez, and of the South-west.* By John W. Monette, M. D., of Washington, Mississippi.

(*Continued.*)

### EPIDEMIC YELLOW FEVER AS IT PREVAILED IN THE SOUTHWEST IN THE SUMMER AND AUTUMN OF 1839.

9. *Alexandria.* This town is situated on the south bank of Red River, just below the rapids, and about 365 miles by the river from New Orleans, or a run of 48 hours by the steam packets. It is the head of steamboat navigation on the lower Red River, during low stages of water. The population generally amounts to about one thousand souls.

The health of this place had never been more uninterrupted in the summer season, than it was for three weeks after yellow fever had been epidemic in New Orleans. The regular steam packets made each their regular weekly trips to and from New Orleans. When the stage of the river admitted the passage of a steamboat over the rapids, they ascended as far as Natchitoches.

The health of the bayou settlements south and east, at the distance of a few miles, was partially interrupted by a few cases of bilious fever. Yet Alexandria continued quite healthy until after nearly a dozen cases of yellow-fever had been introduced by steamboats from New Orleans; besides a number of other persons, who arrived in the meantime, from the same port, apparently in perfect health, and who were soon afterwards attacked by the same disease.

The weather about Alexandria, as well as in all the south-west, had been dry and very hot for weeks together. About the last of July the newspapers of that place complain that "the air at times was almost insupportable"; and that "the town was dry, dull, and healthy," with the mercury in the thermometer ranging from 84° to 92° daily. Such was the state of things in Alexandria when the epidemic was making its first advances in New Orleans; and in a few days more, the atmosphere would have been sufficiently infected to have generated an epidemic there, it being then the head of steamboat navigation from New Orleans, and the point of disembarkation for the swarms of Texian emigrants. An unusual rise or flood in Red River, at this critical juncture, postponed the epidemic for nearly six weeks. Red River continued in flood more or less until late in August, so as to admit the passage of steamboats for that length of time, over the rapids, as far as Natchitoches and farther. That place immediately became the principal destination of the steamboat trade; and Alexandria for the time was relieved of the swarms of emigrants daily ascending to upper Red River and to Texas, as well as of large quantities of freight and goods from the infected district of New Orleans.

So soon as the river subsided to a low stage again, and the rapids were impassable, Alexandria again became the head of steamboat navigation from New Orleans, about the last of August. It was a second time the depot for all the passengers, emigrants, and freight of every kind, shipped for the upper Red

River country. At this time, early in September, the epidemic in New Orleans was beginning to sweep off its scores every day; and every steamboat which arrived at Alexandria introduced more or less *fomites,* besides occasional cases of open yellow-fever, and many persons with the infection dormant in their systems. Cases of yellow fever immediately began to manifest themselves in that part of the town near the river, and in boarding-houses near the steamboat landings. By the 20th of September yellow fever was epidemic in the place; and it prevailed over the whole town before the first of October, although the majority of the people had fled to the country. So strongly infected was the local atmosphere of the place, that several persons from the country, who imprudently visited the town at that time, contracted the disease several days afterwards, and died in the country.

The disease was checked by the cold weather about the first of November, after *one hundred and five souls* had perished. Of these only six were females. The majority of all the deaths and cases occurred among emigrants, boatmen, or transient persons, principally from New Orleans.

Dr. Jackson of Alexandria, and other intelligent gentlemen, assured me that there was not a case of yellow fever in that place until it was introduced by the steamboats from New Orleans. Among the first cases were the engineer of the Houma and some of the crews attached to the Velocipede and Washington steam packets, which run regularly in that trade. The disease first spread from the common boarding houses, where the engineer and other hands of those packets had been left for medical attendance. Those who kept the houses were among the early subjects of the disease.

10. *Natchitoches.* The cases at this place were comparatively few, and were altogether in those who had arrived from New Orleans with the infection in their systems, or were open cases of fever discharged from the boats. These occurred also before the last of August, while the river admitted steamboats over the rapids, and while the epidemic in New Orleans had not reached its greatest degree of virulence. The fall in the river, very opportunely, as the epidemic in New Orleans was approaching its most malignant grade, cut off further intercourse, and this preserved Natchitoches from a fatal epidemic. The Washington packet in her last trip left several persons sick of yellow fever at Natchitoches, who afterwards died. Natchitoches is 150 miles by the river above Alexandria, on the south-west bank of Red River, upon fine ancient alluvion, skirted by rolling pine uplands in the rear; population about 500.

I have not been able to learn that Natchitoches has ever been visited with epidemic yellow fever, as the rapids near Alexandria serve as an excellent natural quarantine ground, during the epidemics in New Orleans.

Having finished our notice of the river towns in which yellow fever prevailed in the autumn of 1839, we will proceed to note a few other points in Louisiana, upon the bayous south of Red River. In several towns of this region, likewise, yellow fever was introduced from New Orleans by steamboats. We desire the reader to bear in mind, that our steamers pass from New Orleans to the distance of 400 miles and return every week; and where the distance is much less, they perform their trips every three or four days. As yellow fever infection lays dormant in the system from three to nine days, before it manifests itself in open disease, persons may travel on steamboats to the distance of 300 and even 500 miles, in perfect health, after they have contracted the disease. If the distance is less they may arrive at their destination several days before the disease developes itself in their systems. There is no part of the southwestern settlements of Louisiana which is more than 250 miles from New Orleans, by the steamboat route; of course the trip to and from the city is performed every week at most, when the business is profitable. At the outbreak of an epidemic in the city thousands of persons leave it within the first three weeks, and disperse through the numerous inland towns within one hundred and fifty miles direct from the city. These points they reach on steamboats in 24 or 36 hours. Under the excitement of anxiety, alarm, and apprehension, the equable condition of the system is disturbed, and many are attacked soon after they reach their destined asylum. Those towns to which many persons, with their families, clothing, bedding, and the like, resort, are greatly exposed to the danger of an epidemic; and especially if the condition of the general atmosphere be favorable to its dissemination. In this manner the yellow-fever was introduced into the following towns in the autumn of 1839:—viz., Thibedeauxville, Franklin, New Iberia, St. Martinsville, and Oppelousas.

1. *Thibedeauxville* is situated on the south side of the Lafourche, about forty miles from its efflux at Donaldsonville, and 125 miles, by steamboat route, from New Orleans. The population is about 300 souls, chiefly Creole French, and some Americans, and transient persons. In low stages of the Mississippi, steamboats cannot pass from the river into the Lafourche, on account of the shoal bottom for several miles of its upper course. At such times the small steamboats which remain in the Lafourche, run regularly from the upper to the lower settlements, a distance of about 100 miles. Donaldsonville then is the depot for the freight and passengers from New Orleans for the Lafourche, and is connected with the Lafourche line of boats by a portage of a few miles. Thibedeauxville is the central town for these settlements, and a regular landing point for steamboats.

In this place the population enjoyed uninterrupted health, in the fall of 1839, until many persons arrived from New Orleans, towards the last of August, for retirement from the epidemic. The *first five or six cases of yellow fever* were unquestionably in persons recently from that city; and none pretend to question the fact, that these cases were introduced from New Orleans. Cases occurred subsequently during the month of September, until the whole number was about *twenty-five.* Some of the latter could not be traced to New Orleans, but to infection or *fomites* introduced from that city. The disease did not prevail as an epidemic, for the greater number of cases were contracted in New Orleans.

Mr. W. B. Shields, an intelligent planter in that region, assures me of these facts. The number of deaths in this town was about fifteen.

2. *Franklin.* This town, like the last, is situated on high alluvion, on the Teche, at the head of steamboat navigation in low stages of the river. The distance from New Orleans by the steamboat route, is about 250 miles. There is also a more direct route in high stages of the river. The trade by steamboats with New Orleans continues all the year. Population 250.

This town, like all the interior towns, was uncommonly healthy during the summer until after yellow fever had become epidemic in New Orleans, and many persons flying from that disease, had arrived from the city about the first of September. In addition to which, in the first week of September, a steamboat arrived from New Orleans with many persons on board, several of whom were attacked soon afterwards with yellow fever, besides *two cases* which developed themselves on the way, one of which died before the boat reached Franklin. This boat proved to be infected; for several persons died who had not been exposed to any other source of infection, and who were attacked with yellow fever a few days after having made a visit to this boat. The clerk of the Parish court was one of them. In less than a week after this boat arrived at the landing, several persons in that immediate vicinity took the disease and also died. The disease was considered epidemic after the 15th of September, and did not cease until checked by frost early in November. The number of deaths in the village and vicinity, was about 25; the whole number of cases about 45.

3. *New Iberia.* This town, like the last, is situated on the Teche, and upon high ancient alluvion, about 30 miles above Franklin, and at the head of low tide-water. The population is about 160.

This village continued very healthy until about the 10th of September, when cases of yellow fever began to present themselves in the persons of those who had recently arrived from New

Orleans, by way of the Plaquemines. Such were the first cases of yellow fever in New Iberia, in 1839. I am not apprised that the disease was ever there before.

Soon after the first cases of this kind, the disease began to spread among those of the place who had been exposed to no other source of infection than the steamboats, the sick, and the *fomites* imported in those boats. The whole number of *deaths*, in and near this town, was about *twenty*. For the facts I am indebted to the Hon. B. G. Tenney, and other intelligent men.

4. *St. Martinsville* is also on the Teche, and about 80 miles by the bayou above Franklin. This village has been noted for its general salubrity, and yellow fever was unknown there until 1839. Several cases occurred, as was the case at Natchitoches, in persons who had arrived from New Orleans; but being in a great measure cut off from a continued supply of cases and *fomites*, by low water, it did not become epidemic in this village.

5. *Oppelousas* is a small, scattered village, situated in a rolling prairie, proverbial for health, and remote from all those causes which are said to generate yellow-fever miasm. It is remote from any navigable stream, and the nearest steamboat landing is nearly six miles distant. With this point, however, the village has direct and constant intercourse by a regular line of daily stages; and thus a direct communication is kept up from New Orleans to Oppelousas. The route between the two points is made in less than 48 hours: a boat may leave New Orleans, loaded with freight and passengers, together with yellow-fever *fomites*, and in 48 hours one half of the whole cargo, and all the passengers, may be in Oppelousas. The intercourse is not discontinued during the whole year; and previous to the experience of 1839, it was supposed, even by those who were well convinced of the importable nature of the disease, that the interruption in the route by a land portage of six miles, was a sufficient guaranty of safety.

This village has long been a resort for many of the inhabitants of New Orleans, when compelled by the epidemic to retire from the city; and in no case have I been able to learn that yellow fever has been ever communicated in its epidemic form, to the inhabitants, until the autumn of 1839. This year the health of the place was uninterrupted, until the first of September, when cases of this disease began to manifest themselves in the persons of those who had recently arrived from New Orleans. For ten days it was confined exclusively to the people of New Orleans, and those recently returned from that city, with whom the village was thronged. Cases multiplied daily, and by the middle of September it was considered as epidemic, when most of the people deserted the place. The disease was epidemic until November, when the number of deaths had increased to *forty-seven*, of whom seventeen were natives of the place.

An infected atmosphere was created, and *first*, especially in some houses where cases had occurred. Some persons from the country, in perfect health, contracted the disease and died after having made a short visit to those houses, supposed to be infected.

Nor was the dissemination of yellow fever from New Orleans, restricted to the inland towns of Louisiana and Mississippi. It was carried likewise to Texas. The principal port of Texas is Galveston, about 500 miles by the sea route from New Orleans. This route is made by the regular packet steamboats in less than 60 hours. Thus, in two days and a half, a steamboat loaded with freight and passengers from the infected port of New Orleans, will be discharging the same at the wharves of Galveston. This kind of intercourse is almost daily during the whole business season. From Galveston to Houston is a drive of eight hours by the stage; and the intercourse is direct and uninterrupted. The port of Galveston has little or no direct trade with any foreign port except New Orleans—it is situated upon an island of sand and of course not in a miasmatic atmosphere, according to the common doctrine. Yet it did become infected with yellow fever as an epidemic, and many lives were lost. Others died with yellow fever at Houston, who left New Orleans only 8

or 10 days before. Galveston received the seeds of the disease from New Orleans, and from New Orleans alone. I am well aware that the Galveston epidemic has been ascribed to the magic influence of marsh miasm, city filth, and impure wharves. It began about the wharves and shipping, we admit, as it does at every port.

If we examine into the circumstances of its prevalence on the northern coast of the Mexican Gulf, from New Orleans eastward to St. Augustine, and even to Charleston, we shall find abundant reason to infer that in the more important ports, yellow fever was introduced from Havana, Vera Cruz and other ports in the West Indies; while in the smaller trading towns it was introduced from New Orleans, Mobile and Charleston. All the commercial ports suffered from the disease in direct proportion to the amount of direct trade with the West Indies, and the smaller towns suffered in due proportion to the direct intercourse with these infected ports of the United States. Other towns, whose latitude and situation, according to the common views of the yellow-fever miasm, would be most exposed to this disease, were entirely free from it, when their intercourse was cut off with the infected ports.

1. *Bay of St. Louis.* This is a small village situated upon the bay of that name, about 60 miles east of New Orleans. This village is entirely without commerce or intercourse with any foreign port, and is made up chiefly of cottages and villas occupied by wealthy families from New Orleans in time of epidemic yellow fever, and at other times as a pleasant summer retreat from the heat and crowded population of the city. The site was chosen for this purpose as one peculiarly free from all the usual sources of disease of all kinds. It is upon a flat sand beach remote from marsh vapors and exhalation, and has always been considered as remarkable for health, and as such, has been a frequent resort for invalids from the interior of Louisiana and Mississippi. The cottages and villas are extremely neat and airy, spread chiefly in one range of buildings at the distance of nearly fifty yards from each other. Besides these there is an extensive hotel for the accommodation of those who have no permanent provision for a residence. There is one steamboat wharf, but no trade or commercial intercourse with any port except New Orleans, and of course none of the usual accumulations of city filth.

Yet when this village has been much frequented and thronged by individuals and families from New Orleans, with constant intercourse by steamboats with the city during the epidemic, it has been repeatedly visited also with yellow-fever cases; and occasionally an infected atmosphere has been generated in one or more houses, from which the disease has been occasionally propagated to those who had been exposed to no other source of infection; and the people have been compelled once or twice to desert the place for safety. The first cases of yellow fever at any time occurring here, have been unquestionably those who had retired from the epidemic in New Orleans or Mobile. In 1839 there was a number of such cases here; but an infected atmosphere was not generated which would produce the disease in those who had been free from any other exposure. What local accumulations of filth, or what marshes are here to generate yellow-fever miasm?

2. *The Bay of Biloxi* is about 30 miles further east, and about 50 miles west of Mobile Bay. It is situated upon the margin of the sea, and like the last, is formed chiefly by about 30 cottages and elegant villas, strung along the beach for several hundred yards. This place is one of common retreat for the two cities of Mobile and New Orleans during their epidemic visitations. Scarcely a season passes, in epidemic years, without several cases of yellow fever, developed in those who arrived apparently in perfect health from one of these infected cities. Yet, like St. Louis, it has been mostly free from an epidemic form of disease. The simple reason for this is, that the local atmosphere in these places is not sufficiently contaminated by a crowded population to form a suitable nidus for the reception of the infection or *fomites.* These facts are given upon the authority of G. L. C. Davis, Esq., an old resident of New Orleans familiar with these places, and it is corroborated by the authority of hundreds. In 1839 seven cases of yellow fever occurred at Belman's Hotel near the steamboat wharf, and the local atmosphere of the Hotel became infected, as was evident to all interested. Several persons contracted the disease from that house.

3. *Mobile City.* This city has an extensive commercial intercourse with the West Indies and other parts of the world. The direct intercourse between Mobile and tropical ports is no more restricted than that of New Orleans, except in point of commercial importance. Besides this it has constant intercourse by steamboats and sea vessels with New Orleans. Owing to its own direct trade with the West Indies, it becomes infected with yellow fever almost simultaneously with New Orleans, while those towns which derive their infection from either, or both, are not visited by the disease epidemically for several weeks after it has prevailed in one or both of these cities. The disease in Mobile is no less fatal than it is in New Orleans. The average mortality in both is at least *one-third* of the whole number of cases; whereas in inland towns the mortality seldom falls short of *one half*, notwithstanding the assertions of some men to the contrary.

The disease in 1839 made its appearance among the shipping at Mobile, simultaneously with the same disease in New Orleans. In both places, the first cases were among the shipping exclusively, or among those who had frequented certain vessels. At Mobile the disease was advancing *pari passu* with that in New Orleans until the 20th of August, when a south wind sprung up for several days, and the disease, for a while, appeared to be arrested. But by the *third* of September it began to spread with great virulence, and extended to the inhabitants of the city. It continued to spread among them for nearly six weeks, with great mortality, until the 20th of October, when it began to abate after a strong wind and change of weather. On the first of November the disease was considered extinct. The total number of deaths was about 650—of these, 147 died in August, 383 in September, and 120 in October. On the first and second days of September there were 22 deaths; and 127 in the *next seven* days.

4. *Pensacola* is situated upon a bay of the same name, about 20 miles east of Mobile, and nearly in the same latitude; but having no West India commerce, it escaped yellow fever. Although this town had suffered severely in former years, from epidemic yellow fever, yet in 1839, when every commercial port was ravaged by that pestilence, Pensacola escaped. Why was Pensacola exempt? Its population was far more numerous than many towns of Louisiana which suffered severely; it is in the same latitude of towns infected, and as far south as Mobile. Why then did it escape, if yellow fever be endemic on this coast? We answer, that Pensacola escaped yellow fever this year, simply because it was not introduced there as it had been in other towns and ports. Pensacola was not crowded with persons who had already imbibed the infection; the town, the stores, and houses, were not filled with goods, clothing, beds and the like from infected towns or ports; it did not carry on a free and extensive commerce with infected ports of the West Indies, with arrivals almost daily of ship-loads of infected air from Havana and Vera Cruz. The streets were not thronged with raw Europeans or unacclimated strangers. These are the negative causes of her exemption.

Pensacola is a naval station, and during the summer and autumn of 1839, the harbor contained a large number of vessels of war, with their complement of sailors and marines. During the greater part of this time, there were at least 30 vessels of war, with their aggregate complements of 3500 men, besides the ordinary population of the place, giving an aggregate of at least 5000 souls.* Yet on the 31st of August, at the very time when yellow fever was ravaging New Orleans and Mobile, the whole population of Pensacola, including the fleets, was perfectly healthy.

During the epidemic season the United States squadron lost five men by yellow fever, having contracted the infection before entering the harbor. The French frigate La Gloiré, en-

---

*See Pensacola Gazette, Aug. 31.

tered the harbor with twenty cases of yellow fever on board. Due precaution was taken to prevent intercourse with the population of the town; the frigate was anchored out in the bay, and the sick were carried to the Navy Hospital, for medical aid. The disease was circumvented and it disappeared.

The French frigate had not become infected, as the air on board had not been contaminated with the infected air of the West India ports, by lying for weeks at the wharves, as merchant vessels do; the men had contracted the disease by going ashore at such places, and the vessel was prevented from being infected by the early removal of the sick to the naval hospital. The disease did not spread at the hospital, because, as we have before remarked, the air in a hospital is not prepared for the dissemination of the disease, like the air of a crowded town or city. The restricted intercourse between the population and the naval forces, prevented the extension of the disease in the town.

The first epidemic in Pensacola, after it passed into the possession of the United States, was in the summer of 1822, or one year after it had ravaged St. Augustine. Up to that period it had been remarkable as a place of extraordinary salubrity. But immediately after the influx of northern emigrants, a trade sprung up between this town and the West Indies, and the yellow fever was introduced. Mr. Williams narrates the facts, and after yielding to the vulgar prejudice, in admitting that it was partly caused by filth and putrescent matters near the harbor, he proceeds to say: "*the pestilence spread like wild-fire*, immediately after the distribution among the huckster shops of the cargo of spoiled fish which *arrived from Cuba*, sweeping whole families, and often whole streets, in one general destruction." The mortality, he informs us, was altogether among the recent population; "for *none of the old inhabitants* were afflicted with the pestilence."*

Here it would appear evident that the active poison which produced the epidemic was certainly brought *from Cuba*, even

* Williams' Florida, pp. 15, 16.

if it were in the "*spoiled codfish.*" Like most other cases of a similar nature, it is likely that the codfish were distributed, sold and eaten, without any discovery of their spoiled condition, until after the close of the epidemic, when probably this plausible conclusion was arrived at. Every shop-keeper who supplied himself with these fish, doubtless *visited the vessel,* and contracted the disease in person, from the infected vessel.

How was it in 1825? Pensacola was thronged with northern adventurers, with trading vessels, and numerous importations for the new settlements, and yellow fever was introduced and prevailed with most destructive mortality. Hence there is nothing in the situation or location of Pensacola, which renders it more exempt from, or more liable to yellow fever, than any other town, under the same contingent circumstances.

The *first* epidemic yellow fever on record at Pensacola, was in 1765, at the close of the French and Spanish war, immediately after a British garrison had taken possession of the province of Florida. "A regiment of soldiers was sent from England to Pensacola, during a very hot summer. On their arrival they were confined day and night within the walls of the fort at Barrancas, which excluded the sea-breeze. They soon became infected with malignant fever, which proved very fatal to the common soldiers."* This epidemic was thus introduced by way of the West Indies, where all the troops of Great Britain were obliged to pass on their destined route. These troops, chiefly fresh from England, with their European constitutions and habits, were brought through the West Indies to Florida, at the very time when yellow fever was epidemic in those seas. They were quartered in a confined fort during the most oppressive heats of summer, under all the circumstances calculated to generate infectious air. This was soon accomplished; and the introduction of supplies of clothing, arms, &c., from the West Indies, furnished them with the active cause of disease, if they did not bring it with them on their first arrival. Those troops in the port, which had no commu-

* Williams' Florida, p. 15.

nication with the garrison in the Barrancas, continued to enjoy perfect health.

Let us inquire into a few other points along the northern shore of the Mexican Gulf. We find Mobile, a commercial city, desolated with yellow fever. At the next bay only 60 miles distant, Pensacola, *not a trading town*, but having at least 3500 men in the harbor, besides her own population, enirely escapes disease; and although twenty-five cases were introduced into the open suburbs of the town, no epidemic supervenes. A little further east, we find the little port of *St. Joseph's*, where a lively trade had sprung up a year or two previously for the new settlements on the lower Apalachicola, with direct intercourse with New Orleans and Mobile, as well as with the West Indies, ravaged by the same pestilence which was then prevailing in those ports. A little further east and south, on the western side of the peninsula of Florida, is Tampa Bay. This point, also, within the last two years had attracted a smart tiading intercourse from the same ports, and after the Seminoles had been driven back, was thronged with settlers and adventurers. This place too became infected, and many of its inhabitants died. Other points were visited in the same manner.

*St. Augustine*, on the eastern side of East Florida, had been proverbial for health during the Spanish regimé, and was never visited with yellow fever until it came under the jurisdiction of the United States. But in the autumn of 1821, having become thronged with northern emigrants and unacclimated adventurers (and mainly on account of its proverbial salubrity), it was suddenly visited by a most fatal epidemic, which swept off a large proportion of all who were attacked.

In 1839, the population of this town was quite healthy until about the middle of August, when yellow fever of a very mild character prevailed to a considerable extent. The place labored under this epidemic from the middle of August until the 12th of November. During this time, out of *seven hundred cases*, only about *fifty* died—or *one* in *fourteen!!* Most cases yielded readily to a judicious mode of treatment. *Yellow fever* never has prevailed epidemically in the United

States, I am persuaded, where there were *more than two recoveries to one death*, including all the genuine cases of yellow fever.

In 1839, I am strongly inclined to think, that St. Augustine was visited with epidemic *bilious* fever, besides a few cases, comparatively, of yellow fever, which may have been communicated from the shipping to those who visited their holds and cabins.

In regard to St. Augustine, the *first epidemic yellow fever* occurred under circumstances similar to those which attended the first epidemic in the Barrancas at Pensacola, viz.: the change of regimé. In the latter it broke out upon the occupancy of the place by British troops by way of the West Indies; in the former, or St. Augustiue, it broke out soon after the place became occupied by the United States. Mr. Williams * states that "in 1821 St. Augustine was visited with yellow fever. It broke out in several buildings situated in the back part of the city, which had been for a long time closed up, their owners having retired to Havana. On the cession of the country to the United States, a *sudden increase of population* caused these houses to be thrown open and *rented to strangers*. One of them was rented to several American officers, and three of them fell immediate victims to the fatal disease. *In some cases the sickness commenced in vessels lying in the harbor*, which had brought fruit *from Cuba. One of these, on the voyage, had lost the captain and most of the crew by sickness. Some early cases of fever were traced to other vessels. . . .* Since that period St. Augustine has been distinguished as one of *the most healthy spots in the United States*." This is a simple, unvarnished narrative, unswayed by prejudice or theory, giving the facts without comment.

6. *Savannah*, the principal commercial port of Georgia, is liable to frequent visitations of epidemic yellow fever. In 1839, the population of the city was uncommonly healthy for at least three weeks after cases of yellow fever began to appear.

---

* Williams' Florida.

We do not design to trace the origin and spread of the epidemic in Savannah, but merely to note the fact, and refer to the circumstance of its appearance among the vessels in the port, several weeks before it became epidemic in the city. The intelligent reader who has perused the previous pages, on the importable character of this disease, can make his own inferences. It first spread from the shipping to the population near the wharves.

7. *Charleston, S. C.*, has been an important commercial port from the earliest settlement under the British Crown. The first epidemic yellow fever in this place, was in the autumn of 1700. It likewise prevailed again in the years 1732, '39, '45, '48, and '92. In more recent times it has been visited occasionally with this epidemic, in its most malignant form. And it is clearly proven by the records of the times, that there have been occasionally cases among the vessels in port, when no epidemic supervened.

We propose briefly to examine the circumstances connected with the prevalence of yellow fever in this city in the fall of 1839, and its *subsequent appearance* at Augusta in the interior of Georgia.

The newspapers represent Charleston as having been remarkably healthy until the first of July. A few cases of yellow fever had occurred among the shipping in port soon after the middle of June; and cases from the vessels were received into the marine hospital on the 25th and 26th of June,* at which time cases were frequent among the shipping. The Charleston Courier, of the 24th of June, 1839, says up to that time, "the cases of yellow fever were few in number, and *confined exclusively to a few vessels* in our harbor, and to *seamen on board those vessels;*" again, "*not a single case had occurred on shore.*" The same paper again declares, that, up to the 24th of June (1839), "among the vast number of *persons now in our city*, liable to yellow fever, *not a landsman has been*

* See Dr. Strobel's Report as Physician to the Marine Hospital. See also the Charleston Courier and New Orleans Bulletin.

*attacked by it.*" The number of cases began to increase daily among the shipping, and chiefly among the crews who were unacclimated. During this time the papers of Charleston continued to repudiate the doctrine which admits the possibility of yellow fever being in any case contagious, or in any wise communicable from the sick to the healthy population; they endeavored to calm the fears of the people against all possible danger, as it possessed, in their opinion, no more communicable properties than bilious or intermittent fever. In this manner they made themselves responsible for the death of hundreds. Many, relying upon such advisers, neglected the necessary precautions; intercourse with the infected vessels was indirectly encouraged, and by this means it was soon disseminated among the contiguous population; and in a few days more it was epidemic among the people residing near the wharves adjacent the infected vessels.*

On the 10th of July the Charleston Mercury urged in very strenuous terms the necessity for "captains and owners of vessels" to be prompt in procuring medical aid, in the *first* attack, while the mass of the citizens were, in effect, encouraged to introduce the disease into the city. The laboring classes as usual, and those who carry on the commercial transactions with the shipping, were the first victims among the inhabitants. The disease continued to spread slowly from the shipping to the population adjacent to the wharves, until the last of July, when it was admitted to be epidemic in the city.†

It is not my purpose in this or any other case, to point out the particular vessel which introduced the disease, because I believe that many are concerned in its introduction; it is the work of some days or weeks, and each vessel from an infected port, has its special agency in inducing an epidemic. There were vessels in the port of Charleston, with yellow fever on board, as early as the 7th of June. The brig Burmah

---

* See New Orleans Bulletin, Aug. 10, 1839, quoted from N. Y. Express.

† Charleston Mercury.

is the first that we can refer to; the brig Braganza was soon after there with yellow fever on board, and many others subsequently.

All agree in the fact that for fifteen days after the disease began to excite alarm, it was confined exclusively to the shipping, and such as came from West India ports. Yet the public press, without evil design, we are sure, endeavored to bolster up the belief that it was a local disease, and a mere grade of bilious fever, produced by local atmospheric influences, although the resident population was at first exempt; while those who were least exposed to such influences, if they had existed, were the only victims. But blinded by an interested prejudice, they would not see.

As soon as the people perceived that the pestilence was extending the sphere of its action, they immediately adopted the usual and only measure for safety, and fled to the country. The rail-road to Augusta offered the most convenient and direct conveyance, and accordingly many embraced the opportunity of retiring to that high and healthy interior town, in the State of Georgia, *where yellow fever had never been known.* Some took with them beds, bedding, goods, and the articles usually taken by retreating families and merchants.

The disease was epidemic in Charleston from the first of August until the 20th of October, when it began to decline in virulence. The number of deaths I have not been able to procure.

8. *Augusta, Ga.* This city, within a few years, has become an important *commercial* depot for importations made through Charleston and Savannah for an extensive region of country in the interior of Georgia and South Carolina. It is also the point at which a great portion of the interior cotton crop of these two States is collected for those two maritime ports. As such depot it is the head of steamboat navigation on the Savannah river; and in 1839 was the *terminus* of the great Charleston rail-road. It is situated upon the Savannah river, 230 miles above the port of Savannah, and 120 miles by the rail-road from Charleston. The site is chiefly tertiary upland formation, in a high and healthy region, beyond the influence of marsh miasm, and all those matters to which yellow fever epidemics in maritime ports are ascribed.

Previously to the summer of 1839, it was proverbial for its salubrity, and noted for its *exemption* from yellow fever during all the ravages of that disease in Charleston and Savannah. Such had been its exemption from this disease at such times, that it furnished a plausible argument against the probable *importability* of the disease; for if importable at all, it might have been at some time transported from Charleston or Savannah, with which constant intercourse was maintained. As such, the fact was used by professor Dickinson of Charleston, in his remarks upon the *yellow fever* of that city in 1838. When those remarks were penned early in the summer of 1839, little did the learned professor think that nature herself would so signally confute the argument and vindicate truth and the immutable laws of nature; that even before his "remarks" met the public eye, she would snatch from him the argument which had been fallaciously used by many others in a similar manner.* But like a philosopher and a votary of scientific truth, he received the lesson taught by nature and renounced his error.†

---

*See Med. Journal published by John Bell, M.D., Philadelphia, December 1839.

†In a letter to Dr. Strobel, of Charleston, dated January 14th, 1840, and published in the Charleston papers, and re-published in the New Orleans Morning Advertiser of December, 1841, Professor Dickinson acknowledges his change of opinion relative to the *importable* nature of yellow fever infection; viz—"I believe yellow fever to be *transmissible or communicable from one city to another*, provided the general circumstances of the two are similar or analogous; that is, I believe the unknown and *obscure cause of yellow fever is transportable from place to place, and in a variety of modes.* This cause requires for its efficiency an undefined occurrence of favorable circumstances, without which it will fail to produce its specific influences; but this is true of the agency of every cause of disease.

Of its contagiousness (using the word in its limited and popular sense), its direct propagation from one subject to another, I have never witnessed an example, and *until recently* should have denied the possession of this property in our climate. *The events of the last summer*,

Augusta remained uncommonly healthy for fifteen days after yellow fever had been epidemic in Charleston, and for nearly ten days after it had been epidemic also in Savannah. Within a few days after the general alarm and flight from Charleston, cases began to appear in Augusta, and chiefly in those who had retired from those infected points, until about the 15th of August. On the 17th and 18th of August, cases began to multiply, and on the 20th it was considered epidemic. From the 18th to the 31st of August, the deaths were only twenty-eight. From that time until the last of September the disease raged with great mortality, after which it began to abate. The number of deaths in the month of September was reported at one hundred and seventy.

The history of this epidemic will show that Augusta was exposed to a double source of infection; first, by the rail-road from Charleston, upon which the train of cars arrive in eight or ten hours; and secondly, by steamboats which reach Augusta in thirty-six hours from Savannah. The former makes daily trips, and the latter are successively arriving once or twice a week, with every variety of articles which retain the infected air in them.

The continued arrivals upon the cars, of persons with the latent seeds of yellow fever in their systems, to be soon developed in open yellow fever, besides other modes of introducing infection, tended strongly to accelerate the epidemic constitution of the city air for leading on the epidemic.

As soon as the inhabitants of Augusta were convinced that they were invaded by this unwelcome visitation, they immediately fled from the city and took refuge in the surrounding country. The physicians of the place, who believed in the local origin of yellow fever epidemics, as usual, began to search

---

however, have inclined me to entertain an *opposite opinion*. An impartial perusal of the statements and arguments of Pym, Blane, Arejula, Wistar, Hosack and Monette, has satisfied me that it deserves to be ranked among contagious diseases.

I am sir, respectfully, your obedient sev't.

B. B. Strobel, M. D. Saml. Henry Dickinson"

for the usual imaginary sources of the miasm. It would havebeen strange if they had not been able to discover some innocent collection of animal or vegetable matter, which would serve, with the credulous, as a scape-goat to bear off the sins of the commercial members of the community who had been instrumental in its introduction, while it diverted the attention of the intelligent from the true cause of the disease. Accordingly the press soon announced that they had discovered "a pile of putrescent vegetable and animal matters near the upper trash-wharf," which no doubt had at once caused all the mischief, although it had remained there in a harmless state since the first of June!

Why did not yellow fever manifest itself in Augusta at the same time it made its appearance in Charleston and Savannah? Why was the epidemic in Augusta deferred until Charleston and Savannah were almost deserted by their inhabitants and the transient population? Why did it not commence in Augusta first, if it proceeded from local causes? Did the arrival of the people from those infected cities excite the extrication of pestiferous miasm from local causes, which had laid dormant up to that time?

We have shown already that an infected air is often generated or imported in vessels arriving in our ports from the West Indies, and that persons have beyond doubt, contracted yellow fever by visiting such vessels after they had arrived. The same atmosphere is likewise generated in steamboats from infected towns. Cholera has in like manner been introduced into many towns within the valley of the Mississippi, as I have abundant evidence to prove, during and subsequent to the Black Hawk war, on the upper Mississippi, several years ago. A cholera atmosphere may be generated from a number of cases on any steamboat or vessel, or in any house or town. The same is true of yellow fever under favorable circumstances in the local atmosphere of any given town.

Professor Dickinson, before cited, observes: "*Cholera is importable.* It may be conveyed from one city to another *by a vessel;* or it may originate during an unfortunate voyage on board a vessel, which shall not only disease her passengers, but will infect persons who may venture on board in perfect health, after her arrival in port. In either case, the vessel herself, or the atmosphere which she contains, shall be in a state capable of communicating the disease to those who visit her. Cholera is, therefore, clearly a proper subject for quarantine regulations, and these should be strict and perfectly effectual."*

This is precisely the case with yellow fever during the hot and oppressive weather of summer in the southern part of the United States. The whole weight of impartial testimony sustains the position; and as it has been known to produce the most malignant and terrific epidemics in the ports and towns of the United States, we deem it a most important and proper subject of quarantine regulations for the protection of our citizens.

This point we shall endeavor still further to sustain and elucidate by facts and reasoning, which we desire to submit to the impartial reader and the candid enquirer after truth.

### ORDINARY MODE OF INTRODUCING INFECTION.

The manner in which infection is introduced into towns and cities on the lower Mississippi, is through the medium or agency of ships or steamboats, as we have already shown in the early portion of our remarks. New Orleans is the point from which it is communicated to the interior river towns; and this city itself, in like manner, is annually supplied with a due portion of *fomites*, and infected air from West India ports, imported chiefly in trading vessels, which arrive almost daily from all those ports where yellow fever is known to be endemic at nearly all seasons of the year.

When any town or city, and especially those south of lat. 35°, contains *infectious air*, as already illustrated, that place is in a *proper condition* for the dissemination of *yellow-fever infection*, and for the prevalence of a fatal epidemic, although

*See Am. Jour. Med. Sciences, 1833, vol. 13, p. 359-60.

the population may have been entirely healthy immediately previous to the introduction of such infection. If this condition of the local atmosphere exists in an eminent degree, a smaller amount of infected air, or *fomites*, will generate the epidemic; if this condition of the local atmosphere is less concentrated, a larger amount of infection or fomites is requisite to produce the same effect; and a town or city in the latter condition may escape an epidemic, after the introduction of an amount of infection, which, under other circumstances, would have generated an epidemic. This position we desire to be borne in mind, for it will prevent the dissenting reader from urging those objections which are nugatory to one who carefully weighs this point.

Hence the arrival of infected ships, and steamboats, with goods on board impregnated with infection, in any port or town, may be eminently dangerous, or it may be comparatively harmless, according to the circumstances of the case. The number of steamboats on the waters of the Mississippi trading to New Orleans is not less than *three hundred.*

All who are practically conversant with yellow fever in the southern States, know full well, that beds, blankets, clothing, and all other porous articles, which remain a short time in a strongly infected atmosphere, whether limited to a single room, house, or ship, or to the mercantile part of a city, become impregnated with the active *materies morbi*, and will produce the disease in those who are subsequently exposed to its operation. It is equally well known that the entire atmosphere in a room will become so thoroughly infected, under certain circumstances, as to produce the disease in divers individuals who may enter such room, and breathe its atmosphere even for a short time.

Let us suppose a case. Yellow fever is epidemic in New Orleans; it begins to carry off its victims at the rate of 50 or 60 per day. Regardless of the danger, hundreds of strangers remain in the city, vainly hoping to escape the disease; one ship after another, crowded with foreign emigrants, German or Irish, arrive in the desolated city, and take up a habitation in some refuse or desolate houses and sheds. Lured by the high wages for labor, they remain and expose themselves in the infected district, until the disease spreads rapidly among them. Every day diminishes their numbers, and a new focus of infection is generated in their camp or lodgement. Finding that the pestilence increases its ravages daily, without a prospect of abatement, they determine to escape from the city and pass on into the interior. They engage passage on the first steamboat for St. Louis or Louisville. The boat has been lying at the wharf in the infected district for several days, taking in freight of every description, and all more or less impregnated with infection. At length the boat is ready for her departure, and our foreign emigrants are crowded into the lower deck to the number of 50 or 100 souls, men, women and children, a motley crowd, packed and stowed, with their filthy bedding and clothes, into every recess and corner of the boat. They continue to sicken and die in this crowded atmosphere, during the hot sultry weather of August or September; and occasionally the boat lands to take in wood, and to bury two or three deck passengers who may have died the last night. The whole atmosphere in that part of the boat, and every article of bedding, clothing, and the like, becomes thoroughly infected, and will produce the disease in those who visit the boat at the different landings on her way up the river. By the time the boat reaches St. Louis or the mouth of the Ohio, fifteen or twenty of this miserable crowd have been laid in quiet repose along the solitary banks of the Mississippi.

This is not a case entirely of fancy; the parallel complete has been too often witnessed on the Mississippi within the last 20 years.

In many cases boats in this condition, on their ascending trip, land at Natchez or Vicksburg, to deposit their sick and dying, and perchance to bury their dead. They relieve themselves of these encumbrances at Natchez, or at Vicksburg, or some other town on the river.

It would be strange, if a succession of boats, in this condition, arriving every day or two, and generally discharging their sick and a part of their foreign emigrants with the disease dormant in their systems, should communicate no disease in the crowded and stagnant atmosphere of the river towns. Yet because very often one or two houses are first infected, or because a particular district of a town or city becomes first infected, we find some of the medical faculty, as well as the illiterate, adduce this very fact as an argument in support of the extrication of a pestilent miasm from *some local cause* which they cannot indicate.

In all these instances the disease first manifests itself in those parts of towns and ports where the above species of population mostly congregate, whether near the wharves or not. However they generally find their temporary residence near the wharves and steamboat landings, in some of the numerous crowded wooden tenements of those places.

The number of German and Irish emigrants that arrive at New Orleans every summer is almost incredible; and of late years it is the principal port for which they take shipping, on account of the facilities afforded for reaching the northwestern States on the upper Mississippi. These crowd the deck of every steamboat which leaves New Orleans for the upper country during the summer, but especially when the epidemic begins to rage in the city.

The constant arrival of these northern foreigners in July, August, and September, at New Orleans, and many of them touching on their way at West India ports, contribute materially to aggravate the malignity of the disease in that city, and to extend it into interior towns.

(TO BE CONTINUED.)

