# Correction

**Published:** 1842-07

**Authors:** 


Correction.—Dr. Monette has requested us to make correction of an error that crept into the MSS. of his last article on yellow fever, published in our preceding number, the entire edition of which had passed through the press before his letter was received. On page 407 (vol. 5, no. 6), instead of the first sentence of the third paragraph, substitute the two following:

"5 Opelousas is a large town or city, beautifully spread over an extensive area, upon a dry, rolling prairie, and proverbial for health. The houses are not crowded, and the streets are spacious and cleanly, and destitute of all the causes which are said to generate yellow-fever miasm."

